# Dental Colleges

**Published:** 1882-12

**Authors:** A. Berry


### Dental Colleges.

BY A. BERRY, D.D.S.

Read before the Mad River Valley Dental Society.

The founders of our dental colleges, Harris and Hayden of the Baltimore, in 1843, and Taylor, Rogers, and Cook, of the Ohio, six years later, did great service to our profession. Prior to this time those desirous of becoming dentists were usually superficially taught by practitioners, and most of them engaged in practice after a few weeks, or months of study. There was no dental school, nor any associated effort in the world for the elevation of the profession. Each member kept his secrets, or imparted the knowledge of any improvement he might make, or became cognizant of, for a consideration.

But this state of things was to be changed, and the first movement to accomplish it was to educate those entering the profession, imbueing them with a love of it, and leading them to associated effort for its elevation. The course adopted was successful. The early colleges did a noble work. The unselfish devotion of their professors was instilled into their students, and in a few years dental societies were formed in various parts of the country. These dental schools succeeding well, others were founded, until they are in every section of our land.

The pioneer colleges received with open doors all pupils who applied, without any regard to their educational status. This was, at that time, perhaps, the best course to pursue. Our's was a new country, and teaching dental students in colleges an experiment; and the fraternity was to be advanced to a state of enlightenment entitling it to take rank with the learned professions.

There are grave objections to the management of most of our dental schools; while it is not to be denied that the teachers engaged in them are the best men for their places. The fault lies in the administration of the colleges.

Formerly there were those who willingly made great sacrifices, as teachers in the dental colleges, believing that, in the state of the profession at that time, it was their duty to do so without financial reward. But now, in the advanced condition of the profession such inducements do not exist, and similar sacrifices are not to be expected. The teachers must now receive financial compensation for their labors. The schools not endowed must hold out strong inducements to attract students. Since they supply the funds it is necessary to matriculate them without requiring them to understand even the common branches of an English education.

So far as the writer of this has been able to learn, a similar course is pursued by the dental colleges connected with universities, excepting those of Michigan and Vanderbilt.

Ichabod might be inscribed on the doors of any one of these colleges, not connected with universities, that would not cheerfully receive all who apply, and graduate them without much regard to their attainments. Students would go elsewhere, and the colleges would die of inanition.

As most dental students, when matriculated, are wanting the mental training to make them apt scholars, and few of them have much knowledge of the studies to be pursued, the time of tuition in our dental colleges is too short. The average term of those, not departments of universities, is about nineteen weeks. Deducting two weeks to get the pupils well at their studies at the commencement of the terms, two weeks for the holidays, and one week for the examinations at the end of each term, leaves fourteen weeks for study. It is not to be presumed that the students will devote much time to study during the vacations.

Are twenty-eight weeks, with all the advantages a college can afford, a sufficient time to acquire a knowledge of anatomy and oral surgery with dissections, physiology, histology, pathology, therapeutics, chemistry, and operative and mechanical dentistry? The result is that most of the students in the dental schools acquire a smattering of what is taught, and are usually graduated without much attention given to the evidences of their attainments, shown by their examination. Is this the proper way to elevate and maintain the standing of a profession recognized as learned, and a branch of that engaged in the practice of the healing art?

There is little probability that dental schools, not connected with universities, would agree on and abide by any rule requiring a good English education of those received as pupils, and extend the time of pupilage to enable them to gain the requisite knowledge of the subjects taught. Were all dental colleges departments of universities, these difficulties might be more readily avoided. Supported from the common fund they would be free from the strong inducements to receive students without preliminary examination, and grant diplomas to those not well qualified.

The interests of the profession as well as of the student, would be greatly promoted by extending the time of lectures in the dental schools to nine months in the year, and requiring attendance on three terms previous to examination for the degree.

